# Micropathogen Community Analysis in *Hyalomma rufipes* via High-Throughput Sequencing of Small RNAs

**DOI:** 10.3389/fcimb.2017.00374

**Published:** 2017-08-15

**Authors:** Jin Luo, Min-Xuan Liu, Qiao-Yun Ren, Ze Chen, Zhan-Cheng Tian, Jia-Wei Hao, Feng Wu, Xiao-Cui Liu, Jian-Xun Luo, Hong Yin, Hui Wang, Guang-Yuan Liu

**Affiliations:** ^1^State Key Laboratory of Veterinary Etiological Biology, Key Laboratory of Veterinary Parasitology of Gansu Province, Lanzhou Veterinary Research Institute, Chinese Academy of Agricultural Sciences Lanzhou, China; ^2^College of Veterinary Medicine, Gansu Agricultural University Lanzhou, China; ^3^Jiangsu Co-Innovation Center for Prevention and Control of Important Animal Infectious Diseases and Zoonoses Yangzhou, China; ^4^Centre for Ecology and Hydrology, Natural Environment Research Council (NERC) Wallingford, United Kingdom; ^5^Department of Engineering, Institute of Biomedical Engineering, University of Oxford Oxford, United Kingdom

**Keywords:** micropathogen community, ticks, *Hyalomma rufipes*, high-throughput sequencing, small RNA

## Abstract

Ticks are important vectors in the transmission of a broad range of micropathogens to vertebrates, including humans. Because of the role of ticks in disease transmission, identifying and characterizing the micropathogen profiles of tick populations have become increasingly important. The objective of this study was to survey the micropathogens of *Hyalomma rufipes* ticks. Illumina HiSeq2000 technology was utilized to perform deep sequencing of small RNAs (sRNAs) extracted from field-collected *H. rufipes* ticks in Gansu Province, China. The resultant sRNA library data revealed that the surveyed tick populations produced reads that were homologous to *St. Croix River Virus* (SCRV) sequences. We also observed many reads that were homologous to microbial and/or pathogenic isolates, including bacteria, protozoa, and fungi. As part of this analysis, a phylogenetic tree was constructed to display the relationships among the homologous sequences that were identified. The study offered a unique opportunity to gain insight into the micropathogens of *H. rufipes* ticks. The effective control of arthropod vectors in the future will require knowledge of the micropathogen composition of vectors harboring infectious agents. Understanding the ecological factors that regulate vector propagation in association with the prevalence and persistence of micropathogen lineages is also imperative. These interactions may affect the evolution of micropathogen lineages, especially if the micropathogens rely on the vector or host for dispersal. The sRNA deep-sequencing approach used in this analysis provides an intuitive method to survey micropathogen prevalence in ticks and other vector species.

## Introduction

Micropathogens pose serious threats to the health of humans, livestock, and wildlife. In many cases, the spread of disease is mediated through micropathogens residing within arthropod vectors. Ticks are haematophagous ectoparasites of both domestic and wild animals as well as humans. These small arachnids can transmit micropathogens, including viruses, bacteria, protozoa, and fungi (Jasik et al., 2015; Jahfari et al., 2016). Ticks and the micropathogens that they transmit cause direct damage to animals by reducing animal weight, milk production and leather quality. Ticks can also cause paralysis, toxemia, and anemia (Hawlena et al., 2013) in animals, thereby hindering the development of a viable livestock industry in tropical and subtropical regions where significant economic losses can occur due to vector-transmitted disease agents (Süss, 2011).

Several reports pertaining to the transmission biology of *Hyalomma rufipes* ticks in China have been published. These reports have provided information relating to the transmission of diseases, such as Crimean–Congo hemorrhagic fever, bovine anaplasmosis and tick typhus (caused by *Rickettsia* spp.), all of which are particularly important in relation to human health and the livestock industry (Estrada-Peña et al., 2006; Hornok and Horváth, 2012; Hüe et al., 2014). Previous studies, although restricted by limited sample sizes, have described the compositions of entire microbial communities in ticks, fleas, and lice involved in the transmission of disease-causing infectious agents (Jones et al., 2008; Rynkiewicz et al., 2015). Similar microbial community analyses have also been described in other blood-feeding invertebrates, such as mosquitoes and leeches (Lindh et al., 2005; Worthen et al., 2006). However, there have been no reports to date on the micropathogen communities in haematophagous parasites and their transmission of infectious microorganisms.

In addition to interactions with coexisting bacteria, microbial agents must also interact closely with their vectors and hosts to facilitate propagation. These interactions are critical as they affect the evolution of microorganism communities in the host, particularly if the associated microorganisms rely on the vector or host for dispersal. Furthermore, some lineages are passed vertically from female vectors to their offspring, and these lineages likely coevolved with the vector to facilitate their maintenance in host populations. Interestingly, some studies have reported that resident bacterial populations can even positively influence host fitness (Kwiatkowski and Vorburger, 2012). Vertically transmitted microbes can also affect insect health, thereby affecting community assemblages (Teixeira et al., 2008).

Many factors are believed to influence the microorganism composition and diversity in parasitic vectors. A recent study by Swei and Kwan (2017) reported that the microbiome composition of *Ixodes pacificus* ticks (the vector for Lyme disease in the western United States) is specifically affected by the identity of the host blood meal. A separate study performed by Zolnik et al. (2016) investigated the microbiome composition in *Ixodes scapularis*. The authors observed that the microbiome composition and the associated bacterial diversity are affected by the developmental stage of the tick vector. Many of the bacterial genera observed in these analyses are associated with the environment in which the tick species is found. This environmental impact on the microbiota was also observed for the Rocky mountain wood tick (*Dermacentor andersoni*), and the authors suggested that the microbiome composition might be important in vector susceptibility to environmental contaminants (Clayton et al., 2015). Interestingly, the symbiotic relationship between tick and pathogen was also highlighted by Abraham et al. (2017) when they described a non-passive role for pathogens in the vector microbiota, demonstrating that pathogenic bacteria critically affect vector gene expression, thereby readying the vector to facilitate pathogen infection by manipulating the microorganisms in the vector. The importance of these interactions between resident microorganism populations and vectors prompted us to further investigate the microorganism communities and their diversity in field-collected *H. rufipes*.

Small RNAs have been shown to play crucial roles in gene functions for most biological processes, including cell differentiation, proliferation, and metabolism (Chu and Rana, 2007; Filipowicz et al., 2008; Kim et al., 2009; Morris and Mattick, 2014). For this reason, the regulation of small RNAs is a key approach in experimental biology to understand the mechanisms of biological phenomena at the molecular level in microorganism communities. Thus, far, several types of microbial communities have been identified by various sequencing platforms, including sampled disease vectors, animal guts, and various microenvironments (Jones et al., 2010; Rosenthal et al., 2011; Bowman et al., 2012; Laport et al., 2017).

To explore the microorganism communities in parasitic vectors, we used high-throughput sequencing to identify the community compositions in wild-caught *H. rufipes* ticks in Gansu Province, China. From a community ecology perspective, this approach allowed us to identify the diversity of putative micropathogen agents, the presence of which could subsequently be confirmed by reverse transcription polymerase chain reaction (RT-PCR). Importantly, to the best of our knowledge, this is the first comprehensive survey of the entire micropathogen community in a tick vector known to participate in the transmission of parasitic agents. This study is also the first to explore the utilization of a high-throughput technique to identify the composition of micropathogen communities in a tick vector that facilitates parasite transmission. In this study, we describe a method for the detection of micropathogen communities. We also describe the prevalence of resident micropathogens within a disease vector, and we propose that tick microbiome monitoring has potential as a strategy to help us better understand ectoparasitic disease vectors.

## Materials and methods

### Tick collection and RNA extraction

Ticks (*H. rufipes*) were captured across Yongjing County, Gansu Province, China, from April 15 to July 23 in 2011. They were identified at the Department of Veterinary Parasitology, Lanzhou Veterinary Research Institute, Chinese Academy of Agricultural Sciences. To remove external bacteria, the collected ticks were immediately placed in phosphate-buffered saline (PBS) and washed twice in a solution containing 0.133 M NaCl, 1.11% sodium dodecyl sulfate (SDS) and 0.0088 M EDTA. Fifty clean ticks were mixed and stored in liquid nitrogen until the ticks were crushed, and the samples were then subjected to lysozyme treatment for 30 min at 37°C (lysozyme facilitates the lysis of bacterial cells and optimal RNA recovery). After lysozyme treatment, RNA was extracted using a Total RNA Purification Kit (TaKaRa, China) following the manufacturer's recommended protocol.

### Small RNA sequencing

The quality of total RNA was analyzed with both a Shimadzu 206-97213C BioSpec-nano analyzer system and a denaturing polyacrylamide gel electrophoresis system. A small RNA library was generated according to the Illumina sample preparation instructions (Chen et al., 2012). The RNA fragments were reverse transcribed using M-MuLV (Invitrogen) with reverse transcription (RT) primers (as recommended by Illumina) to generate single-stranded cDNA. The cDNA was subsequently amplified with Pfx DNA polymerase (Invitrogen) using 20 PCR cycles and the Illumina small RNA primer set. PCR products were purified, and the recovered cDNA was precipitated and quantified with both a NanoDrop spectrophotometer (Thermo Scientific) and a TBS-380 mini-fluorometer (Turner Biosystems) using the PicoGreenH dsDNA quantitation reagent (Invitrogen). The sample concentration was adjusted to 10 nM, and 10-mL final volumes were used for the sequencing reaction. The purified cDNA library was used for cluster generation (on the Illumina Cluster Station). The cDNA was subsequently sequenced on an Illumina HiSeq2000 machine, following the manufacturer's instructions.

### Standard small RNA analysis

The raw sequencing reads contained some low-quality sequences. To create reliable data for analysis, we processed the raw reads as follows: (1) eliminated low-quality tags (the criteria are listed in the explanation of each row in Table 1); (2) eliminated tags with 5′ primer contaminants; (3) eliminated tags with no 3′ primer; (4) eliminated tags without insert; (5) eliminated tags with poly As; (6) eliminated tags shorter than 18 nt; (7) summarized the length distribution of the clean tags; and (8) identified the rRNA, tRNA and snRNA molecules following alignment using Rfam 10.1 and the GenBank database. The results are shown in Table 2. The clean read data were assembled using the SOAP *de novo* short sequence assembly software (http://soap.genomics.org.cn/soapdenovo.html). The program and parameters were -k 17, -d 1, and −r 2, and −M indicated a control for the mismatch number. The resultant clean tags were mapped to the assembled results using bowtie (http://bowtie-bio.sourceforge.net/manual.shtml). Reads that could not be mapped were once more assembled using the SOAP software. The assembly was repeated twice until contigs > 50 bp in length could not be assembled. Unique contigs were then obtained by merging several assembled contigs and removing redundancies (Supplementary Data Sheet S1). Here the merging contigs is how sequence clusters are assembled from various samples, and these clusters are merged to together.

**Table 1:** The sequencing chromatogram is converted into sequence data during the base calling step.

**Term**	**Description**
Total reads	Total sequenced reads, which must be > 5 M in general (except for serum samples)
High-quality	Number of high quality reads with no Ns, no more than 4 bases with quality scores lower than 10 and no more than 6 bases with quality scores lower than 13
3′ Adaptor null	Number of reads with no 3′ adaptor sequence
Insert null	Number of reads with no insertion
5′ Adaptor contaminants	Number of 5′ contaminants
Smaller than 18 nt	Number of reads < 18 nt; generally, small RNA tags are between 18 and 30 nt long and therefore, tags that are too short should be removed from the data prior to further analysis
Poly A	Number of reads with Poly As
Clean reads	Number of clean reads after adaptors and contaminants are removed that are used in subsequent analyses; detailed information for the clean reads has been submitted to the manuscript by Supplementary Data Sheet S1

*In addition, per a standard, sequencing quality is assigned*.

**Table 2:** The data is processed by the following steps: (1) removal of low quality reads (the criteria for this step is listed in the explanation of each row in Table 1); (2) removal of reads with 5′ primer contaminants; (3) removal of reads without a 3′ primer sequence; (4) removal of reads without the insert tag; (5) removal of reads with poly As; (6) removal of reads shorter than 18 nt; and (7) summary of the length distribution of the clean reads.

**Type**	**Count**	**Percent (%)**
Total reads	13756626	
High quality	13736581	100
3′ Adapter null	11255	0.08
Insert null	3236	0.02
5′ Adapter contaminants	29681	0.22
Smaller_than_18 nt	36234	0.26
PolyA	186	0.00
Clean reads	13655989	99.41

*In addition, the numbers are shown in this table*.

To identify small RNA candidates, we analyzed RNA libraries of 15- to 40-nt small RNA molecules. A library was generated from *H. rufipes* ticks and mapped to annotated small RNA loci after discarding the adapter sequences to detect micropathogens in the ticks. The tick small RNA was removed from the sample library using two protocols: (1) for small RNAs from species with information in miRBase18, the small RNA tags were aligned to a genome database of the corresponding species in the NCBI database with parameters of blastall -p blastn -F F -e 0.01 and (2) for small RNAs from species with no information in the NCBI database, the small RNA tags were aligned to the NCBI genome database of all plants and animals with software developed by BGI, tag2miRNA. In addition, to identify the sequence source, i.e., micropathogen or tick, all of these small RNA tags were aligned to the NCBI genome database of all plants and animals.

### Microbial sequence detection using blast and PCR confirmation

BLAST searches were conducted with ncbi-blast-2.2.26+ to identify micropathogen sequences in the clean unique reads. Due to the large amount of high-throughput sequencing data, we formatted the sequencing reads (using formatdb, which is included in the BLAST package) based on the BLAST database and used the micropathogen sequences downloaded from the EMBL website (http://www.ebi.ac.uk/embl/) as query sequences to expedite the BLAST process. The BLAST results were then analyzed manually to screen for potential viral, bacterial, fungal, and protozoan sequences.

To verify the reliability of the data pertaining to microbial community composition in *H. rufipes* following high-throughput sequencing, we first identified the microorganisms in *H. rufipes* by PCR. These micropathogens have been previously described and included *St. Croix River virus* (*SCRV*), *Candidatus Midichloria mitochondrii* (*CMM*), Crimean-Congo hemorrhagic fever agent, *Anaplasma marginale, Rickettsia conorii*, and *Babesia occultans* (Attoui et al., 2001; Epis et al., 2013; Aktas et al., 2014; Gazi et al., 2016; Ionita et al., 2016; Capelli-Peixoto et al., 2017). Total tick cDNA was extracted using a RevertAidTM First Strand cDNA Synthesis Kit (TaKaRa, China) according to the manufacturer's instructions. The primers were selected from several typical microbial sequences assembled with mappable reads. The PCR reactions were performed in an automatic DNA thermocycler (Bio-Rad, Hercules, CA, USA), and the PCR products were separated by 1.5% agarose gel electrophoresis to assess the presence of specific bands indicative of micropathogens. The DNA fragments generated were recovered and ligated into the pGEM®-T Easy vector (Invitrogen, Carlsbad, CA, USA) and then used to transform competent *Escherichia coli* DH5α cells (Takara Bio Inc., Dalian, China). At least three positive clones were sequenced per sample by the GenScript Corporation (Piscataway, New Jersey, USA).

### Phylogenetic and taxonomic analysis

The *rpoA* gene of *CMM* and the *SN2* and *vp6* genes of *SCRV* were randomly selected for the phylogenetic tree. Multiple sequence alignments were performed using CLUSTALW (http://www.ebi.ac.uk/clustalw/). Then, a neighbor-joining (NJ) phylogenetic tree was constructed using MEGA software version 5.0, and the branching reliability was tested using bootstrap re-sampling (1,000 pseudo-replicates).

### Real-time qPCR

To validate the distribution characteristics of the micropathogens in ticks collected from the field, we conducted RT-qPCR using micropathogen-specific primers (listed in **Table 4**). The amplification conditions were as detailed above. A RevertAid First Strand cDNA Synthesis Kit (Thermo. China) was used for RT.

## Results

### Standard small RNA analysis

**Figure 1:**
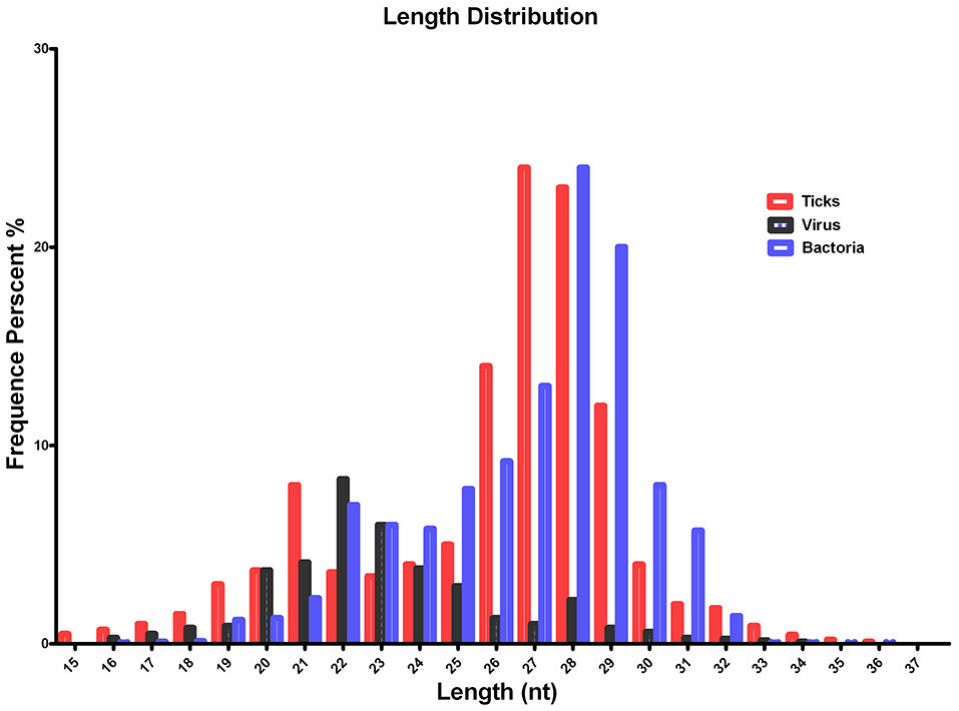
Summary of the tag length distribution following the sequencing and annotation of small RNA from *Hyalomma rufipes*. The length of the small RNAs varied between 18 and 30 nt. Length distribution analysis is helpful when elucidating the composition of small RNA samples. For example, miRNA is normally 21 or 22 nt, siRNA is 24 nt, and piRNA is 30 nt. The x-axis represents the length of the small RNAs. The y-axis represents the percentage frequency of small RNAs of specific lengths among the sequencing reads. Red indicates the length distribution of total clean reads from ticks and the first peak generated by the Dicer enzyme; the second peak was generated by the Piwi enzyme. Black indicates the length distribution of *SCRV*. The peak was mediated by Dicer activity. Blue indicates the length distribution produced by Dicer and Piwi from the bacteria communities. Nt indicates nucleotides.

The quality of the sequenced small RNAs (based on length distribution) is shown in Figure 1; the clean reads were of high quality and in accordance with internationally recognized standards for sequencing (Xu et al., 2010). Two small RNA peaks were identified in the ticks that were analyzed; the first peak was at the 22 nt site, and a second peak was observed at the 28–29 nt site (Figure 1, red). The length distribution of the bacterial small RNAs (28–29 nt) was consistent with the data generated from the tick population (Figure 1, blue). The viral small RNA populations generated only one peak at 22 nt (Figure 1, black), which was relatively short for small RNAs.

**Figure 2:**
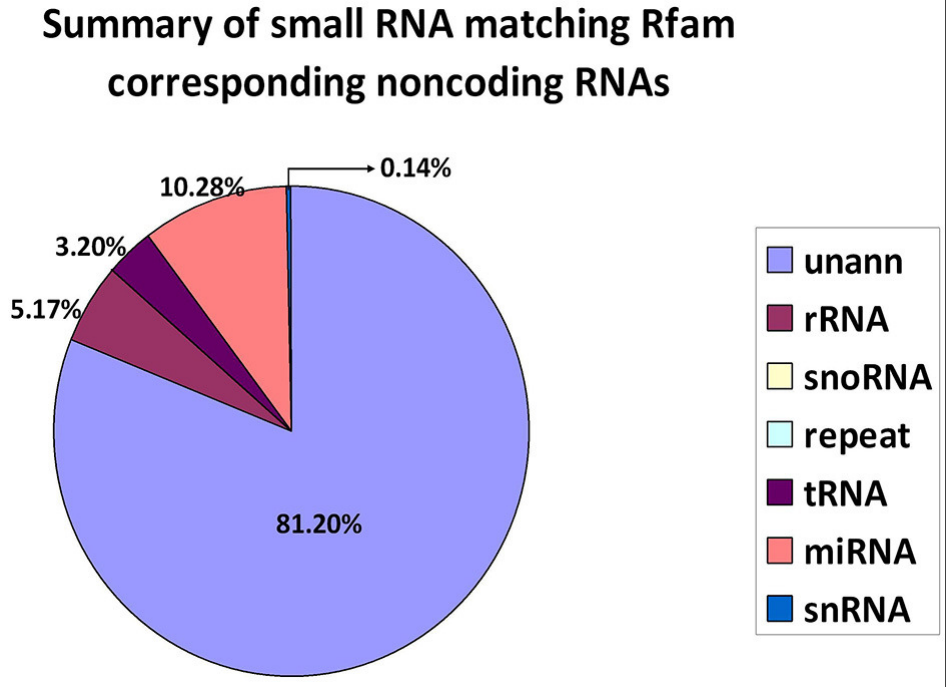
Distribution of small RNAs among different categories. Unann, unannotated; rRNA, ribosomal RNA; snoRNA, small nucleolar RNA; tRNA, transfer RNA; miRNA, microRNA; and snRNA, small nuclear RNA.

The alignment and annotation of these sequences serve as important quality indices for sequencing data. To construct unique small RNA populations that mapped to only a single annotation, we used the following priority rule: RNAs in GenBank > Rfam > known miRNA > repeat > exon > intron (Calabrese et al., 2007). The results revealed that 81.20% of the sequences were unannotated. The miRNA, rRNA and tRNA sequences represented 10.28, 5.17, and 3.20% of the total sequences, respectively (Figure 2).

### Data assembly and bacterial diversity analysis

In total, 13,655,989 small RNA sequences were generated from all of the *H. rufipes* tick samples that were analyzed. These sequences were cleaned following the removal of low-quality tags and other contaminating sequences from the 50-nt tags. In addition, the sequences were assigned to 3,337,930 unique phylotypes. However, most of these phylotypes did not match any genomes in the NCBI database. The sequences resulted in 95,015 contigs with a total length of 7,193,001 bp. The maximum contig length was 901 bp, and homology analysis was performed for these contigs at the nucleotide and protein levels using the NCBI database. The total length and % GC of these contigs were 30,155 bp and 46.51%, respectively, and the majority of the sequences were 27 bp long. These data were then assembled, and any contig > 50 bp was aligned to the nt and nr libraries of viruses, Rickettsiales, bacteria, fungi and parasites with 13.79, 19.58, 16.11, 3.77, and 4.46% of contigs, respectively, found in their roots. The *e*-values and scores were relatively high for *SCRV* and *CMM* as well as for *Bacillus smithii, Anaerococcus hydrogenalis*, and *Caenorhabditis remanei* (Table 3).

**Table 3:** Summary of the bioinformatics data assembly and micropathogen diversity analysis.

**Classification**	**Species**	***E*-value**	**Score**	**Max sub-length (bp)**	**Percent of root (%)**
Virus	*St Croix River virus*	1E-07	60.5	1345	13.79
Rickettsiales	*Candidatus Midichloria mitochondrii*	8E-05	50.8	811	19.24
	*Rickettsia endosymbiont of ticks*	2E-03	46.2	323	0.34
Bacteria	*Escherichia coli*	1E-05	53.3	360	1.38
	*Helicobacter bizzozeronii*	0.003	45.1	299	2.06
	*Rhodospirillum photometricum*	4E-04	53.1	63	0.69
	*Brucella pinnipedialis*	0.002	45.4	50	0.34
	*Streptomyces sviceus*	1E-06	57	50	1.37
	*Vibrio cholerae*	3E-08	62.4	169	0.34
	*Bacillus smithii*	7E-09	48.5	103	2.06
	*Clostridium* sp.	2E-05	64.3	95	0.69
	*Pseudomonas syringae pv*.	2E-05	53.1	94	0.34
	*Corynebacterium efficiens*	2E-04	49.7	261	1.03
	*Acinetobacter* sp.	6E-05	51.2	50	1.03
	*Citrobacter youngae*	1E-04	50.1	109	0.34
	*Legionella pneumophila subsp*.	6E-03	44.7	93	0.69
	*Lactobacillus jensenii*	2E-04	49.3	174	0.34
	*Providencia stuartii*	2E-03	46.2	189	0.34
	*Rhodococcus opacus*	4E-04	48.5	80	0.34
	*Ruminococcus obeum*	2E-05	52.8	40	0.34
	*Alistipes indistinctus*	6E-05	51.2	395	0.34
	*Leptonema illini*	0.001	47	122	0.34
	*Providencia rettgeri*	0.001	46.6	86	0.34
	*Streptomyces* sp.	0.004	45.1	195	1.03
	*Anaerococcus hydrogenalis*	1E-09	67	67	0.34
Fungus	*Trichoderma virens*	0.003	45.1	82	0.69
	*Trichoderma reesei*	1E-06	56.6	383	0.34
	*Trichoderma atroviride*	2E-05	52.8	131	1.03
	*Myceliophthora thermophila*	3E-03	45.8	129	0.34
	*Aspergillus niger*	1E-04	50.4	58	0.34
	*Neurospora tetrasperma*	2E-04	49.7	118	0.34
	*Melampsora laricis-populina*	1E-08	63.5	160	0.69
Parasite	*Trichinella spiralis*	0.008	43.9	920	2.41
	*Caenorhabditis remanei*	3E-07	58.9	1564	0.69
	*Schistosoma mansoni*	4 E-04	48.5	99	0.34
	*Clonorchis sinensis*	6E-06	54.7	1324	0.34
	*Saccoglossus kowalevskii*	3E-05	52.4	141	0.34
	*Brugia malayi*	0.008	43.9	21	0.34

*Scores indicate the homology between sequences; higher scores indicate greater similarity. The e-values indicate the reliability of the evaluation score. The underlining shows that 12 human-infective micropathogens were identified in the ticks*.

The genera and families of the order Rickettsiales were recently reorganized and redescribed by Dumler et al. (2001). Several species from this order were reported to be associated with arthropods, particularly with ticks. Phylotype analysis showed that *CMM* accounted for a high percentage (19.24%) of the assembled sequences from the tick sample reads; these reads matched (100%) the *rpoA* gene from the *CMM* genome (IricVA chromosome, ID: NC_015722). Also of interest was a viral species (*SCRV*) that represented > 13% of the viral community in the tick samples.

### Detecting viral sequences

**Figure 3:**
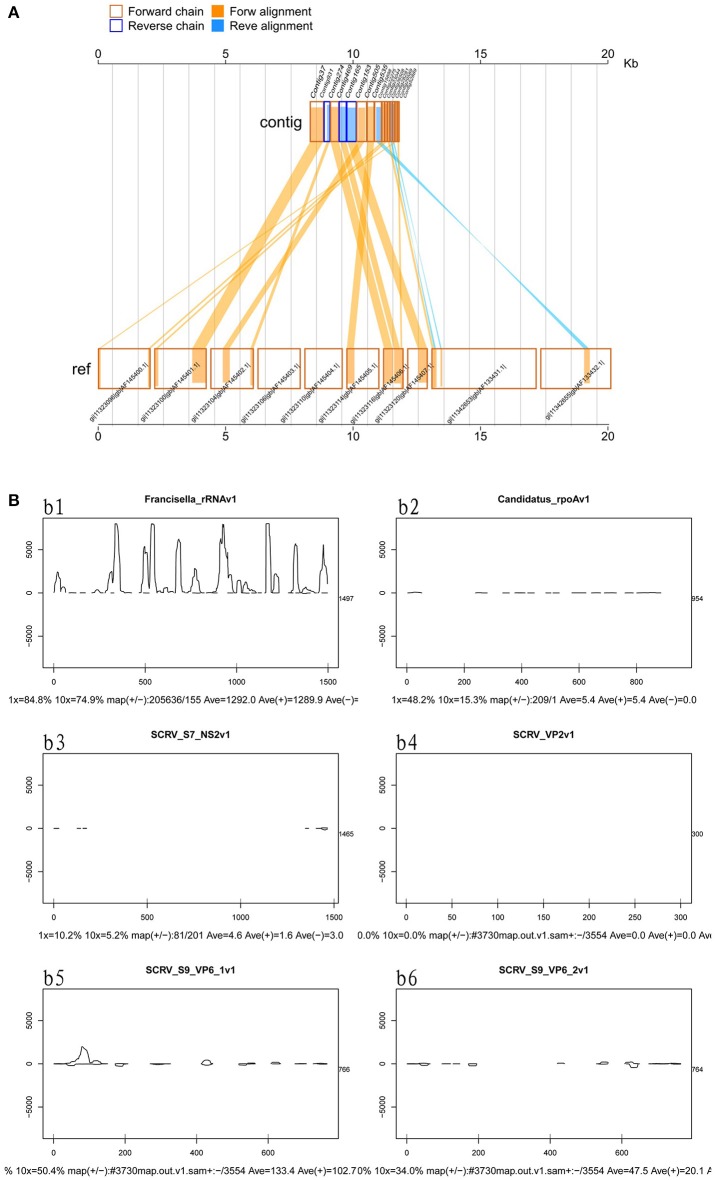
Mapping of micropathogen sequencing reads to genomes. **(A)** Cleaned sequence reads were mapped to the *SCRV* virus genome (GenBank accession number PRJNA14941). The *SCRV* genome contains 10 open reading frames (Vp1, Vp2, Vp3, Vp4, Vp5, Vp7, Vp9, Vp10, NS1, and NS2), which are flanked by inverted repeats and direct repeats at both termini of the genome. Dashed lines represent the regions to which sequenced reads were mapped. Yellow indicates the coverage (occurrence frequency) of the sense strand reads, and blue indicates the coverage of the antisense strand reads. **(B)** Small RNA mapping to the reference genome sequences of the corresponding micropathogens. b1–b6: These figures indicate deep coverage of the virus genome by the small RNA map following use of the pileup format. Both 1x and 10x indicate (yes and no) % coverage, average depth, and the proportion of forward and reverse (+/-) strands. b1: Mapping of the *Francisella* 16S rRNA to the genome (ID: 13795) for the negative control. b2: A 100% match was observed for the *rpoA* gene of *CMM* from *H. rufipes* and the *CMM* IricVA chromosome (ID: NC_015722). b3, b4, and b6: Genes SN2, Vp2, and Vp9 matched (100%) the genome of *SCRV*. b5: The reads from *SCRV* from *H. rufipes* show one mismatch with the reference genome.

Viruses represent extremely important animal pathogens. In this study, we performed BLASTN searches to identify potential viral sequences from the cleaned and unique sequences, revealing that a large number of unique sequences from *H. rufipes* shared identity with one virus, *SCRV* (GenBank Accession: PRJNA14941). Sequence alignment using the Vp6 and SN2 sequences from previously identified microorganisms revealed that the virus identified as part of this analysis exhibited more than 95% amino acid sequence identity with the published *SCRV* sequence (Accession numbers: YP052950.1 and YP052948.1, respectively). To characterize small RNA sequences with homology to viral genome sequences, mapped sequence reads were assembled using default parameters. The results showed that the small RNA reads overlapped, allowing contig assembly, with the longest assembled consensus sequence being 901 bp and the minimum length being 50 bp. Although most of the viral transcripts (Vp2, Vp6, and Vp9) were covered by the viral RNA sequences, the small RNAs were not evenly distributed along the transcripts. Some sites had relatively high coverage, with 3 sites exhibiting very high coverage (Figure 3A). We also utilized a pileup format mapping approach and found that the degradation of *SCRV* and *Francisella* rRNA enzymes were different (Figure 3B). These results indicate that a virus with a genetic sequence homologous to *SCRV* was present in the *H. rufipes* sample analyzed in this study.

### Bacterial infection of *H. rufipes* identified by PCR

To explore the presence of micropathogens in *H. rufipes* ticks, we performed PCR on the samples collected. We investigated the presence of some major micropathogens, such as those responsible for Crimean–Congo hemorrhagic fever, bovine anaplasmosis, tick typhus (caused by *Rickettsia* spp.) and bovine babesiosis using the specific primers listed in Table 4. The results were negative, indicating the absence of these microorganisms in the *H. rufipes* ticks. These results were consistent with the data generated from high-throughput sequencing in which none of the aforementioned micropathogens were observed. We also performed RT-PCR using the total cDNA generated from RNA extracted from the ticks as a template to confirm the presence of micropathogens in our samples. Ten additional primers were successively designed by a walking approach and used in the reactions. These primers are listed in Table 4. Gel electrophoresis of the amplified products confirmed the presence of micropathogen sequences of the appropriate size in *H. rufipes*. Sequencing of the RT-PCR products revealed sequences that were identical to those in our small RNA assembly, confirming the existence of micropathogens exhibiting substantial homology to *SCRV* and *CMM* in the *H. rufipes* ticks analyzed.

**Table 4:** Target genes and list of primers used in this study.

**Micropathogens**	**Primer names**	**Primer sequence (5′–3′)**	**References**
*St. Croix River virus*^a^	ScrvVp6f	5′-ACGCTGGATCGGACATGAA-3′	The primers were designed manually based on the high-throughput sequencing data
	ScrvVp6r	5′-GGGTATGAGAAGAGATGCATG-3′	
	ScrvSN2f	5′-GTAATGCAAGAGATCAGCATG-3′	
	ScrvSN2r	5′-CACCGCCCTGATAAACATACC-3′	
*Candidatus Midichloria mitochondrii*^b^	Cmmf	5′-ATGTATGGTCCAGCTATTGG-3′	
	Cmmr	5′-CCACGGGAACCAATGTACTTC-3′	
*Crimean-Congo hemorrhagic fever agent*	Cchfvf	5′-ATGAACAGGTGGTTTGAAGAGTT-3′	Spengler et al., 2015
	Cchfvr	5′-TGGCACTGGCCATCTGA-3′	
*Anaplasma marginale*	Amaf	5′-TTATGGCAGACATTTCCATATACTGTGCAG-3′	Palmer et al., 2001
	Amar	5′-GGAGCGCATCTCTCTTGCC-3′	
*Rickettsia conorii*	Rcof	5′-GCTCGATTGRTTTACTTTGCTGTGAG-3′	Millán et al., 2016
	Rcor	5′-CATGCTATAACCACCAAGCTAGCAATAC-3′	
*Babesia occultans*	Bocf	5′-GACACAGGGAGGTAGTGACAAG-3′	Decaro et al., 2013
	Bocr	5′-GATCCTTCYGCAGGTTCACC-3′	

a *Viruses and bacteria detected by high-throughput sequencing. The short sequences were assembled to a maximum length of 901 bp, and the resulting sequences were used to design the primers*.

b *Primers designed based on the assembled sequences*.

### Phylogenetic distribution of novel lineages

**Figure 4:**
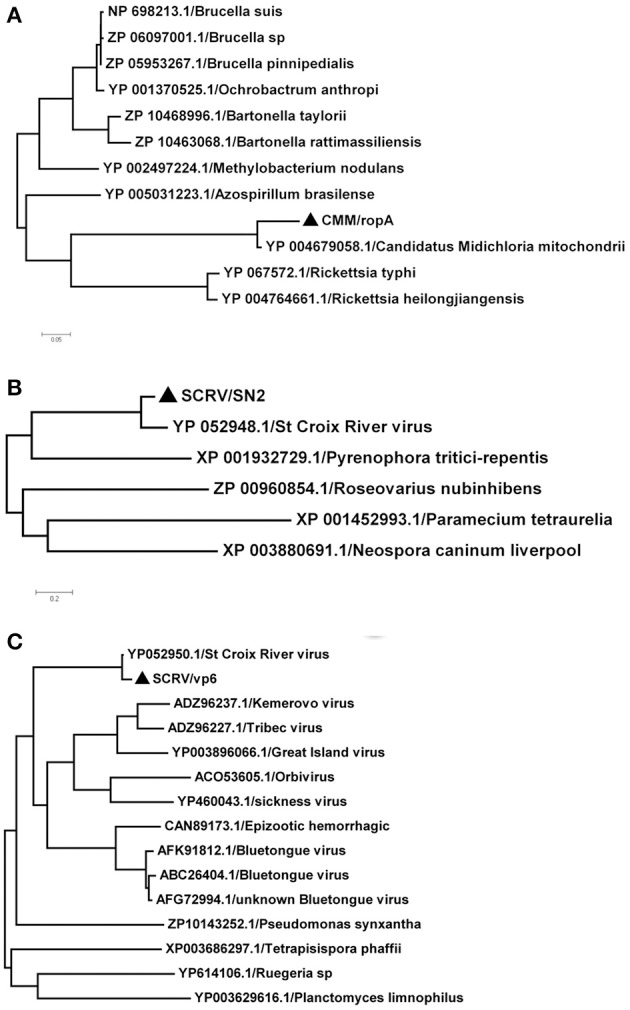
Phylogenetic analysis of the isolated bacteria/viruses. The phylogenetic tree was generated using MEGA 5.0 with maximum parsimony and 500 bootstrap replicates. Reference amino acid sequences were selected by BLAST searches of the NCBI nt database. **(A)** Subtrees of the experimental sequences from the *CMM rpoA* gene. **(B)** Subtrees of the experimental sequences from the *SCRV* SN2 gene. **(C)** Subtrees of the experimental sequences from the *SCRV* Vp6 gene.

In this study, *SCRV* and *CMM* are important pathogens. To analyze the diversity of the putative *SCRV* and *CMM* genomes in the tick populations examined, five different *SCRV*-specific sequences and the *rpoA* gene from *CMM* were amplified and sequenced to construct a phylogenetic tree. The *rpoA* gene indicated a relatively close relationship between *CMM* (identified in the *H. rufipes* populations analyzed) and rickettsiae (Figure 4A). In addition, we observed that the SN2 gene of *SCRV* was closely related to the fungal micropathogen *Pyrenophora* (Figure 4B), while the Vp6 gene shared a common ancestor with Orbivirus and Bluetongue virus (Figure 4C). Indeed, Attoui et al. (2001) previously reported that *SCRV* is related to the orbiviruses.

### Identity of micropathogen sequences amplified from wild ticks

Our sequencing analysis revealed high copy numbers of *SCRV* and *CMM* in *H. rufipes*. As part of this study, we examined wild ticks in the Xinjiang Uygur Autonomous Region (XUAR), which is home to numerous wild tick species, each with the potential to harbor transmittable micropathogens. We collected 180 ticks from XUAR to screen for the presence of *SCRV* and *CMM* via RT-PCR. The results showed that *CMM*-positive amplification of the *rpoA* gene was observed in only 3.33% (6/180) of the ticks analyzed, although the *rpoA* gene from this study was 93% similar to homologous genes in GenBank. However, attempts to identify *SCRV* by RT-PCR amplification of the Vp1 genes were not successful. Thus, it is possible that *SCRV* was not actually present in the ticks sampled from XUAR.

## Discussion

Tick populations are known to harbor many different micropathogens, including fungi, bacteria, viruses and protozoa. The presence of these micropathogens can result in infections of both wild and domesticated animals as well as humans (de la Fuente et al., 2008). Evidence is beginning to emerge suggesting that the relationship between arthropod vectors of infectious agents and resident microbial populations is an important factor that determines whether micropathogen species are transmitted from the arthropod to the host (Finney et al., 2015). Thus, the future identification of complex communities of microbial species via next-generation sequencing may facilitate the development of effective control measures against tick vectors. In this study, we surveyed *H. rufipes* tick populations for tick-borne micropathogens. We believe that these findings will improve our understanding of the factors that affect the transmission of micropathogens by the *H. rufipes* vector. The resultant data will also be beneficial for the analysis of the relationship between ticks and their resident microbial populations.

In this study, we identified micropathogens in field-collected *H. rufipes* samples. The success of the method utilized for micropathogens population analysis proved that the high-throughput sequencing approach is applicable not only for the characterization of arthropod micropathogens but also for the discovery of potentially novel micropathogens. As part of this analysis, we also utilized diversity measurements to explore the interactions between lineages within a community. These approaches helped us to estimate the micropathogen community composition in *H. rufipes* samples. Micropathogen lineages assemble in a non-random fashion in ticks. This non-random assembly also occurs when ticks are partitioned, suggesting that these events are not due to stochastic variation in the phylotype prevalence. These results are consistent with a previous study reporting that flea-associated micropathogen communities are not random assemblages but are instead governed by interactions between micropathogen lineages and the flea's place in space and time (Jones et al., 2010).

To improve sequencing quality and facilitate better alignment and annotation of the small RNAs that were identified, we analyzed the length distribution as part of this analysis. The clean reads of small RNAs from ticks resulted in two peaks, with the first peak emerging at the length of the Dicer enzyme site (22 nt) and the second peak at the length of the Piwi enzyme site (28–29 nt, Figure 1, red). Interestingly, the length distribution for viruses was predominantly shorter than that observed for micropathogens, with only one peak at the Dicer enzyme site (22 nt) (Figure 1, black). The bacterial miRNAs resulted in longer nucleotide reads that fit the Piwi enzyme signature at 28–29 nt (Figure 1, Blue). The total rRNA proportion is indicative of sample quality. For instance, the proportion of total rRNA should be < 60% in plant samples (Hao et al., 2012) and 40% in animal samples (Yuan et al., 2013). In our samples, the proportion of rRNA was 5.17%. This result suggests that this sequencing approach generated good-quality sequencing data that could be used for future analyses.

The remaining sequences were clustered based on sequence similarity, and given the imprecise nature of cleavage with RNase III (Dicer), it is likely that the related sequences came from a common precursor. Upon cluster analysis, we decided that the sequences within a cluster that gave rise to the largest number of reads were likely to be the real sequences due to their relatively high prevalence (Thompson and Pope, 2002); the resultant clusters were subsequently chosen for further analysis. Assembly analysis of the sequences indicated increased diversity within these phyla. However, the lack of diversity at the phylum level corroborates previous suggestions that blood-feeding insects have less diverse microbial communities due to the immune response of the associated hosts and/or the low nutrient quality of blood (Graf et al., 2006).

BLAST searches conducted to identify the 39 microbial gene catalogs in our assembly resulted in the identification of 12 human-infective micropathogens, including *Neurospora tetrasperma, Anaerococcus hydrogenalis*, and *Brugia malayi* (Table 3). Each of these micropathogens can potentially harm humans following parasitic transmission (Song et al., 2003; Bhat et al., 2004; Foster et al., 2005). The micropathogen community that was observed as part of this study also contained *Brucella pinnipedialis, Vibrio cholera, Clostridium sp*. and *Rickettsia endosymbiont*, each of which has known micropathogen potential in animals and humans. Several parasite species, including *Caenorhabditis remanei, Trichinella spiralis* and *Schistosoma mansoni*, were also detected in these annotated sequences, suggesting potential relationships with *H. rufipes*. Future studies will help us to determine how these micropathogens are maintained within the microbial communities and what potential roles they play in the community.

As part of this study, we identified microorganisms in the *H. rufipes* tick that were highly homologous with *SCRV* and *CMM. SCRV*, which is a distinct orbivirus species, was previously isolated from the cells of *Ixodes scapularis* (Attoui et al., 2001). The presence of *SCRV* in tick egg extracts suggests that the transovarial transmission of *SCRV* may occur in ticks (Attoui et al., 2001; Bell-Sakyi and Attoui, 2013). *CMM* is also capable of harming both animals and humans (Skarphédinsson et al., 2005; Mariconti et al., 2012; Gofton et al., 2015) and has previously been shown to be an endosymbiont of the *Ixodes ricinus* tick (Sassera et al., 2006). Our analysis suggests that *CMM*, Rickettsiales and *SCRV* can be transmitted by ticks. Whether these micropathogens are transmitted by other vectors remains to be elucidated. The *SCRV* genome sequences were assembled and mapped to viral genomes that showed significant homology to the amino acid sequences for Vp2, 6, and 9. The results of this analysis suggest that *SCRV* is capable of persistently infecting ticks, thereby promoting both increased transmission to the vector host and vertical transmission within the tick population. Furthermore, elevated levels of the aforementioned structural proteins may help mask the virus from the tick and/or host immune system.

Interactions between bacterial lineages within arthropods have been suggested to alter the virulence and population dynamics of the resident bacteria (Magalon et al., 2010; Bernhauerová et al., 2015; Wood et al., 2016). However, only a limited number of large-scale studies pertaining to bacterial communities in ticks and other tick vectors have been conducted to explore these interactions. A previous study demonstrated that field-collected *Haemaphysalis longicornis* harbors a diverse array of microbial communities (Liu et al., 2013). The diverse nature of these communities suggests the occurrence of complex ecological interactions between host and micropathogens, and the discovery of these interactions will hopefully provide insight into the potential control of ticks (Liu et al., 2013). However, it is worth mentioning that the latter study, which analyzed this phenomenon, permitted only limited detection of non-neutral interactions because only five microbial lineages (as opposed to the entire microbiome) were studied. This present study provides a more accurate estimation of microbial community dynamics and the interactions among all the community members.

The distinct geographical environment and climatic conditions in XUAR may influence the diversity of the resident species. To characterize the potential microbial population characteristics of different tick species from XUAR, we utilized RT-PCR to detect *CMM* and *SCRV* in *Rhipicephalus sanguineus, Hyalomma detritum*, and *Haemaphysalis longicornis* ticks and to detect the causative agents of Crimean–Congo hemorrhagic fever, bovine babesiosis and tick typhus (caused by *Rickettsia* spp.) in *H. rufipes* samples. Approximately 3.33% of the *Rhipicephalus sanguineus* and *Hyalomma detritum* specimens that were analyzed were positive for *CMM*. However, we were not able to detect any genes belonging to *SCRV*. Furthermore, we did not detect any genes from the causative agents responsible for Crimean–Congo hemorrhagic fever, bovine babesiosis or tick typhus (caused by *Rickettsia* spp.) in the wild *H. rufipes* ticks. Conversely, *SCRV* was clearly identified in *H. rufipes* isolated from Gansu Province. The *H. rufipes* sample that we collected is possibly representative of an isolated population or may not have been infected with the micropathogens that were not detected. Consistently with previous reports, this result suggests that these micropathogens might have increased potential to lead to epidemic outbursts, especially where their distribution is high (in regions, such as XUAR and Gansu) (Tian et al., 2012). However, further experiments are necessary to verify that some of these microbial agents can be transmitted in the wild.

In conclusion, our study is the first to explore the application of small RNA high-throughput sequencing methodologies for micropathogen discovery in wild-caught vectors. Our results suggest that small RNA sequencing can facilitate the identification of numerous microbial species in the microbiomes of wild-caught ticks. This strategy obviates the need for either culture-based micropathogen isolation or prior knowledge of the associated etiological agents. Furthermore, the technology used in this study is likely to represent an ideal tool for the surveillance of novel emerging bacterial and viral diseases. This application could also be used to monitor microbial communities in infectious insect vectors. Finally, a more thorough understanding of the ecological factors associated with the prevalence and persistence of micropathogen lineages associated with vectors will ultimately help to predict and prevent the spread of disease.

## Author contributions

JL, GL, and HW conceived the study design and participated in field study and JL drafted the manuscript. ZC, QR, and ZT particpated in data analysis and interpretation. JH, ML, and FW carried out laboratory examinations. JXL and HY revised the draft. All authors read and approved the final manuscript.

### Conflict of interest statement

The authors declare that the research was conducted in the absence of any commercial or financial relationships that could be construed as a potential conflict of interest.

## Supplementary material

Supplementary Data Sheet S1 We removed some of the contaminant reads from the sequencing results to obtain the final clean reads. The clean read data were assembled using the SOAP *de novo* short sequence assembly software.
